# Functional outcomes of proximal row carpectomy: 2-year follow-up

**DOI:** 10.1590/1413-785220152306150054

**Published:** 2015

**Authors:** Luiz Garcia Mandarano-Filho, Débora Schalge Campioto, Márcio Takey Bezuti, Nilton Mazzer, Cláudio Henrique Barbieri

**Affiliations:** 1 Universidade de São Paulo, Faculdade de Medicina de Ribeirão Preto, Hospital das Clínicas, Hand and Microsurgery Group, Ribeirão Preto, SP, Brasil. Work developed at Universidade de São Paulo, Faculdade de Medicina de Ribeirão Preto, Hospital das Clínicas, Ribeirão Preto, SP, Brasil.

**Keywords:** Osteonecrosis, Carpal Bones/surgery, Pseudarthrosis

## Abstract

**OBJECTIVE:**

: To evaluate functional outcomes of patients submit-ted to proximal row carpectomy for the treatment of wrist arthri-tis

**METHODS:**

: This is a retrospective study using wrist motion and grip strenght of patients diagnosed with Kienböck disease and scaphoid non-union surgically treated by this technique

**RESULTS:**

: Eleven patients with 2-year follow-up were evaluated. Wrist motion (flexion, extension and ulnar deviation) and grip strength were significantly better from preoperative values. Ho-wever, no difference in radial deviation was observed in these patients

**CONCLUSION:**

: Proximal row carpectomy provides an alternative option for treatment of wrist arthritis, resulting in better active range of motion and grip strength in the long run. **Level of Evidence IV, Case Series.**

## INTRODUCTION

The proximal carpectomy is a surgical procedure used in de-generative diseases of the wrist that preserves the possibility of movement.^1^ ^3^ Indications are late conditions secondary to injury of the scapholunate ligament (scapholunate advanced collap-se, SLAC); nonunion of scaphoid (scaphoid nonunion advan-ced collapse, SNAC); Kienböck disease; failure of implants to the scaphoid or lunate, and chronic perilunate dislocations.^1^ ^4^ ^5^ Contraindications include chondral lesions in the proximal pole of the capitate or lunate fossa on the distal radius.^6^

The aim of this study was to analyze the functional results (ran-ge of motion and palmar grip strength) of patients undergoing proximal carpectomy in the treatment of traumatic or non-trau-matic degenerative conditions of the wrist.

## MATERIALS AND METHODS

This is a retrospective analysis of patients operated between February 2002 and February 2012, approved by the Institution's Ethics Committee under CAAE number 36705614.7.0000.5440. We evaluated 21 patients undergoing proximal carpectomy. Data were collected from medical records and functional as-sessments of patients by the Hand Therapy team in the preop-erative, immediate postoperative and late postoperative period. The functional evaluation consisted of analogic goniometry of the active range of motion of the wrist (flexion, extension, ra-dial deviation and ulnar deviation) and grip strength measured by a Jamar^(r)^ dynamometer (USA) in the affected wrist and its contralateral side. Flexion and extension goniometry was per-formed with the goniometer's arms on the dorsal side of the third metacarpal and the other on the dorsal side of the radius. For measuring radial deviation and ulnar deviation, the center of the goniometer was placed on the head of the capitate on the dorsal surface of the wrist, one arm on the third metacarpal and the other on the dorsal side of radius.^7^ The measurement of strength was always made with the limb parallel to the upper torso, elbow in 90° flexion, forearm and wrist in a neutral position and the dynamometer set at the second position (specific to assess grip strength).^8^ ^9^ The simple arithmetic mean of three measurements with one minute interval between them, alternat-ing between the dominant and non-dominant side was consid-ered. For comparative analysis, we considered the measures made one week before the surgery and two years afterwards. Statistical analysis was performed using the non-parametric Wilcoxon-Mann-Whitney test for dependent variables, due to the small sample size. *p* -Value was calculated by normal approach.

### Surgical Technique

All patients underwent anesthetic block of the brachial plexus and operated supine over a hand table. Venous drainage was gravitational and with the help of Esmarch bandage. The tour-niquet was set at the arm level.

The access was longitudinal between the third and fourth ex-tender compartments until exposure of the capsule, which was also split longitudinally. The articular surfaces were inspected to investigate possible chondral lesions, especially in the lunate fossa and the proximal pole of the capitate. Resection was always initiated by the lunate and pyramidal, and ended in the scaphoid, which was removed cutting to pieces with a gouge. Care was taken to preserve the volar radiocarpal ligaments, thus preventing possible ulnar translation of the carpal. In no case the distal pole of the scaphoid was left. Likewise, radial styloidectomy and neurectomy of the posterior interosseous nerve were also not performed in any case. After the removal of the tourniquet and hemostasis revision, the capsule was closed anatomically with non-absorbable thread, without bringing in the radiocarpal joint. Any kind of internal or external fixation was ever used. The volar splint, placed in the immediate postope-rative period, was maintained in all cases for four to six weeks, when the movement of the wrist was started gradually, assisted by the Hand Therapy team.^10^

## RESULTS

Of the 21 patients who underwent proximal carpectomy, six did not return for clinical evaluation, three requested discharge from the outpatient facility before completing one year after surgery and one died. In all, 11 patients completed follow-up and were followed until at least two years after surgery for func-tional evaluation. Of these, two were female and 9 male. Four patients had Kienböck disease (stage IIIb) and seven presented non-union of the scaphoid (SNAC). The right side was affected eight times and left, three times. Only in two cases the affected side was not the dominant side. The mean age for the final func-tional evaluation, two years after surgery, was 42.6 years old.

### Statistical analysis

Statistical analysis showed significant differences (p <0.05) in the comparison of wrist flexion pre and postoperatively (*p-* -value = 0.0006); extension (*p* -value = 0.0337); ulnar deviation (*p* =0.0289); grip strength (*p* -value = 0.0006) and when com-paring the contralateral wrist (not affected) and the operated wrist (*p* -value=0.0002). The evaluation of the contralateral wrist grip strength averaged 37.45 kgf, standard deviation 3.8 kgf, minimum 30 kgf, maximum 42 kgf and a median of 38 kgf. There was no significant difference comparing radial deviation before and after surgery (*p* -value = 0.3475).

**Figure 1:**
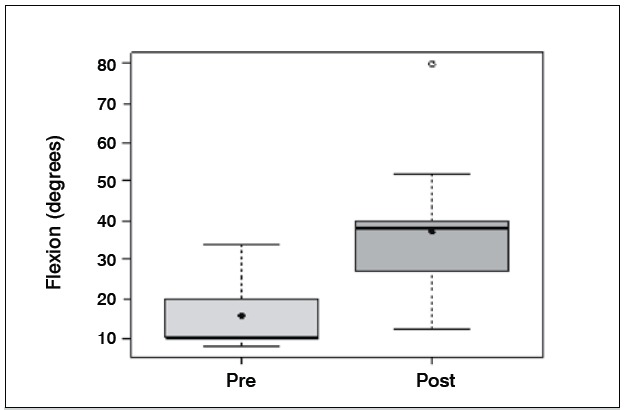
Boxplot comparing flexion of the wrist (degrees) preoperative and two years after surgery

Tables 1-^3^ and Figures 1-^6^ present these data and those regarding measurement (goniometer and grip strength) pre and postoperative.

## DISCUSSION

A Jamar^(r)^ dynamometer (USA), recommended by the American Society of Hand Therapists and used in in most studies was used to assess grip strength. The second prehension position was used for all patients, since it facilitates the comparison of results between them and among other works in the literature. However, there is a possibility of adjusting the position accor-ding to the hand size of each individual.

We adopted a minimum interval of one minute between me-asurements to ensure that there was no influence of the fac-tor muscle fatigue. We used simple arithmetic mean of the three measures. Caporrino et al.^9^ considered the highest mark achieved between the three measures. Probably the difference between these methods is minimal and the important thing is to maintain the same measurement standard before and after surgery.

**Table 1:**
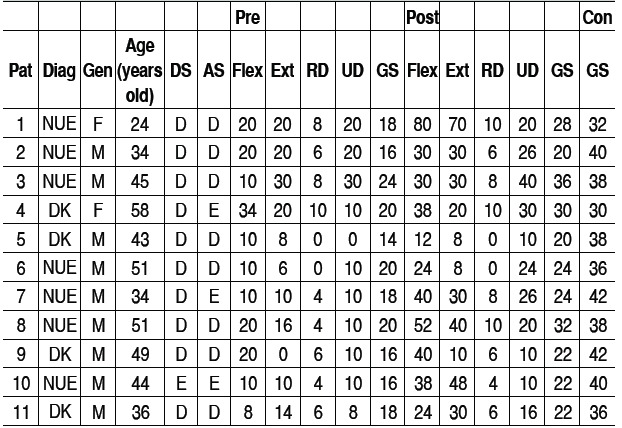
General data and functional results.

Pat: patient; Diag: diagnosis; Gen: gender; DS: dominant side; AS: affected side; Flex: flexion (de-grees); Ext: extension (degrees); RD: radial deviation (degrees); UD: ulnar deviation ulnar (degrees); GS: Grip strength (kgf); Pre: preoperative; Post: postoperative; Con: contralateral wrist

**Table 2:**
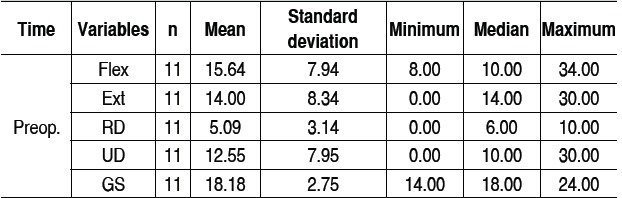
Combined preoperative results.

Preop: preoperative; Flex: flexion (degrees); Ext: extension (degrees); RD: radial deviation (degrees); UD: ulnar deviation ulnar (degrees); GS: Grip strength (kgf).

**Table 3:**
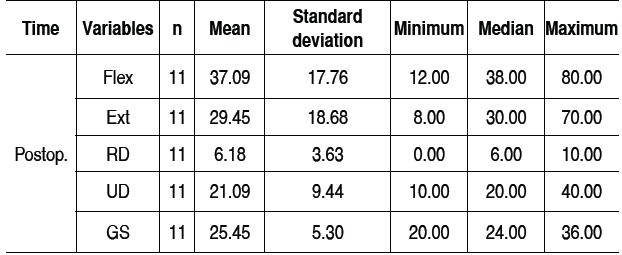
Combined postoperative results.

Postop: postoperative; Flex: flexion (degrees); Ext: extension (degrees); RD: radial deviation (de-grees); UD: ulnar deviation ulnar (degrees); GS: Grip strength (kgf)

**Figure 2:**
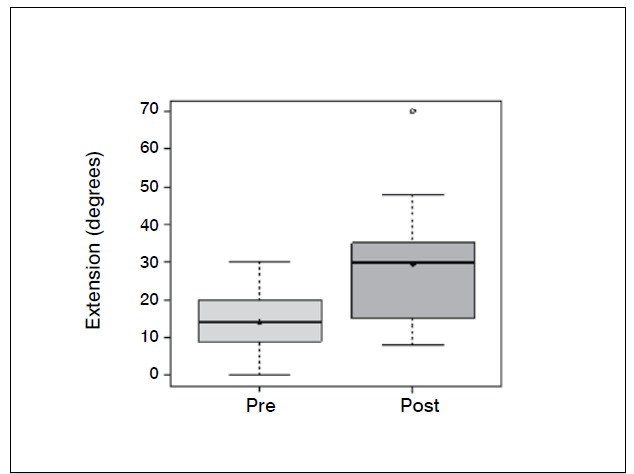
Boxplot comparing extension of the wrist (degrees) preoperative and two years after surgery.

**Figure 3:**
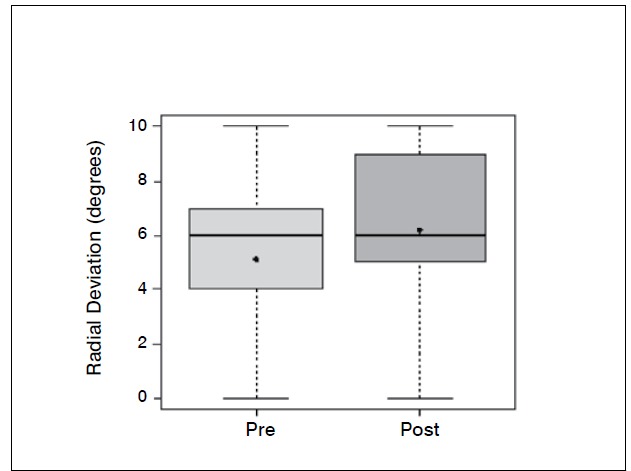
Boxplot comparing radial deviation of the wrist (degrees) preopera-tive and two years after surgery.

**Figure 4:**
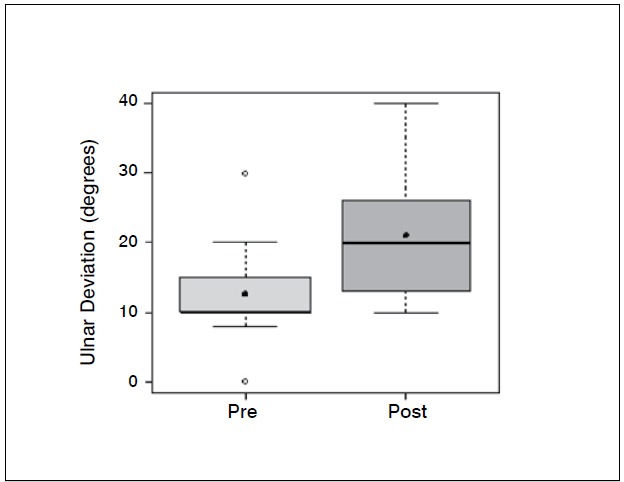
Boxplot comparing ulnar deviation of the wrist (degrees) preopera-tive and two years after.

**Figure 5:**
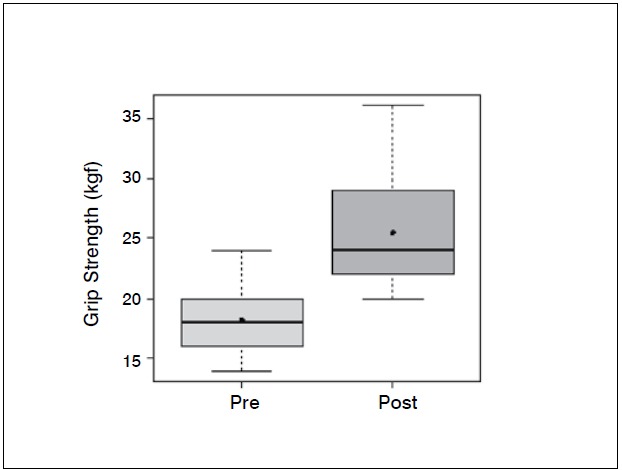
Boxplot comparing grip strength of the wrist (kgf) preoperative and two years after surgery

**Figure 6:**
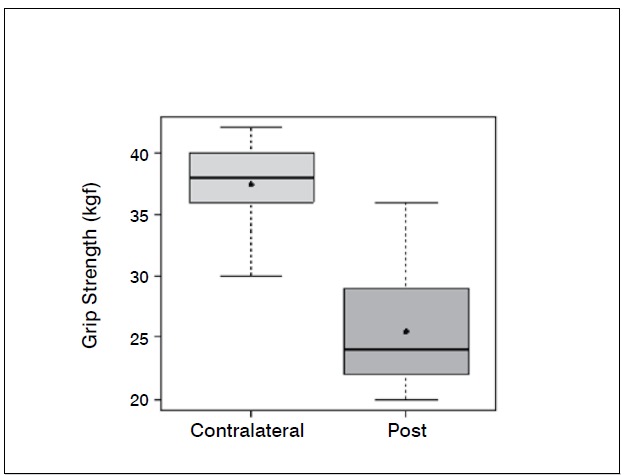
Boxplot comparing grip strength of the contralateral wrist (kgf) preoperative and two years after surgery.

Of the 11 patients who were evaluated after two years of sur-gery, the causative basis of diagnosis of degenerative clinical handle frame was nonunion of scaphoid fractures (SNAC) and Kienböck disease. There were no cases secondary to the injury of scapholunate ligament (SLAC), which could be included in the functional evaluation. Other medical conditions where the proximal carpectomy can be indicated such as rheumatoid arthritis, flexion contractures (congenital or acquired), chronic perilunate dislocations and replantation were not observed, however, they would be excluded from the series according to their unique characteristics that hinder a comparative analysis. The data show that there was a significant improvement in the ability to actively bend (15.6° to 37.0°) and extend (from 14.0° to 29.4°) the wrist, and ulnar deviation (12.5° to 21.1°). There was no significant gain in the radial deviation (5.1° to 6.2°). The largest portion of the population shows flexion between 70-80°, and extension 60-70°, 45° ulnar deviation and 15° radial devia-tion.^7^ The total range of motion for flexion-extension showed an average increase of 29.6° to 66.4°, which represents a much better ability to spatial positioning of the hand and obvious functional gain. The pronosupination and goniometry of passive movement of the wrist were not evaluated in this study. Although not being fundamental parameters in this situation, they could enrich the final evaluation.

Grip strength also improved significantly, from 18.2 kgf in the preoperative evaluation to 25.4 kgf after two years of surgery. The comparison with the unaffected side shows that there is an important difference, since the average was 37.4 kgf. Popula-tion studies in our midst show that the difference between the dominant and non-dominant hand is around 10 to 12%.^10^

Pre and postoperative pain has not been systematically evalu-ated with visual or numerical scales. After two years of surgery, according to medical records, five patients (46%) had no pain or pain was reported as mild and tolerable and went back to work. There is no reference to the type of work or if there was function exchange. Three patients (27%) had pain equal to the preoperative situation, but did not want another surgery. Of these, two returned to work; there is no information about the third. Three patients (27%) had worsened pain relative to baseline, one of which evolved with clinical signs compatible with complex regional pain syndrome. These three cases were subsequently submitted to total wrist arthrodesis.

Our results show correspondence to other studies with high-er postoperative follow-ups; 1 3 11 and comparisons between techniques.^2^ ^5^

Auxiliary surgical techniques can bring improvement in pain symptoms, such as the interposition of the volar capsule,^12^ osteochondral grafts^13^ and posterior interosseous neurecto-my, however, they were not used in this series. Further studies should be conducted to compare the results of the association or not of these procedures, as well as the proximal arthroscopic carpectomy.^14^

Function scores (DASH, QuickDASH, MHQ - Michigan Hand Questionaire^15^ and MWS-Mayo Wrist Score^16^) and radiographic review for staging of osteoarthritis radiocapitate^6^ ^17^ can also be used in future studies as a way to refine the evaluation of results in the medium and long term.

## CONCLUSION

The proximal carpectomy is an alternative in the treatment of degenerative disorders of the wrist, improving range of motion and grip strength, when compared to preoperative status.
